# Supplementary material for: Identification of photoperiod-induced specific miRNAs in the adrenal glands of Sunite sheep (Ovis aries)
Source: Front Vet Sci. 2022 Jul 22;9:888207. doi: 10.3389/fvets.2022.888207 (PMC9354845; doi:10.3389/fvets.2022.888207)
Supplement: Supplementary file 1 [file Data_Sheet_1.ZIP › Supplementary file/miRDeep2 file.zip!/miRDeep2 file/Novel_Structure/SP42A3/Novel_163.pdf]

Provisional ID	:	NC_019480.2_28934
Score total	:	5.6
Score for star read(s)	:	3.9
Score for read counts	:	-1.8
Score for mfe	:	2
Score for randfold	:	1.6
Score for cons. seed	:	
Total read count	:	8
Mature read count	:	6
Loop read count	:	0
Star read count	:	2

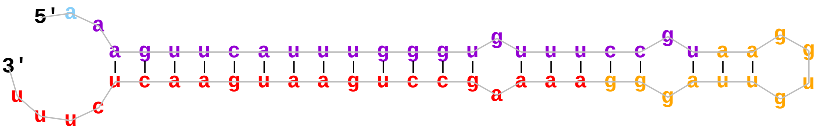

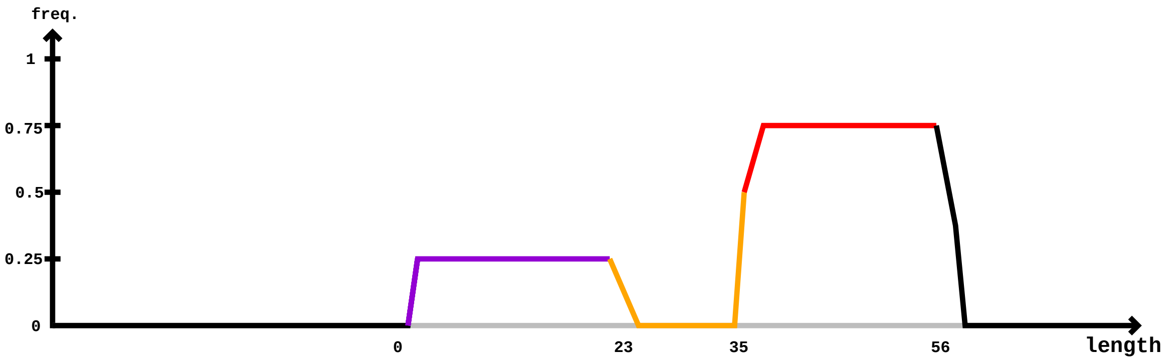

Star Mature

```
5'- agguucuuaccuucuucgugcauugaguuggcuaaaaaguucauuuggguguuuccguaagguguuagggaaaagccugaaugaacucuuuggccaacccaaauauuuaau   -3'    obs
    agguucuuaccuucuucgugcauugaguuggcuaaaaaguucauuuggguguuuccguaagguguuagggaaaagccugaaugaacucuuuggccaacccaaauauuuaau          exp
    ((((....))))..........(((.((((((((((.(((((((((((((.(((((.(((....))).))))).))))))))))))).)))))))))).)))........   reads      mm        sample
    ....................................aaguucauuuggguguuuccgu.....................................................   2          0         seq
    ......................................................................aaaagccugaaugaacucuuu....................   3          0         seq
    ......................................................................aaaagccugaaugaacucuuugg..................   1          0         seq
    .......................................................................aaagccugaaugaacuUuuugg..................   1          1         seq
    ........................................................................aagccugaaugaacuUuuugg..................   1          1         seq
```
